# The Corset Question

**Published:** 1870-04

**Authors:** 


### THE CORSET QUESTION.

"SINCE women have loosened their corsets, the annual mortality has decreased eighteen and a half per cent." Such is the announcement in one of the papers, as coming from the "Medical Press," of which we never heard before. How did the writer find out that women had loosened their corsets? how did he find out how many did that thing? over how large a field did his observations extend? Quite as absurd as that cerebral fevers have increased 72¾ per cent. since chatelains, chignons, and waterfalls have come into use. Why didn't he make it even seventy-five, or three quarters, it would have been so much easier to remember? The truth is, those particular figures were given to throw an air of accuracy around the statement. Any editor, with a thimbleful of common sense, ought to have seen the perfect absurdity of the statement from the very consideration of the impossibility of obtaining statistics on that subject with the means or money of any ordinary man. That corset wearing injures the health, and that the various styles of waterfalls have injurious tendencies, we do not deny; but random statements on any subject pertaining to health and disease are a public injury.

